# Impacted Supernumerary Teeth–Early or Delayed Intervention: Decision Making Dilemma?

**DOI:** 10.5005/jp-journals-10005-1173

**Published:** 2012-12-05

**Authors:** Seema Gupta, Nikhil Marwah

**Affiliations:** Senior Resident, Department of Pediatric Dentistry, Maulana Azad Institute of Dental Sciences, New Delhi, India, e-mail: seema.mds@gmail.com; Reader, Department of Pediatrics and Preventive Dentistry, Mahatma Gandhi Dental College and Hospital, Jaipur, Rajasthan, India

**Keywords:** Supernumerary, Surgical intervention, Early and delayed intervention

## Abstract

Supernumerary teeth are considered to be one of the most significant dental anomalies affecting the primary and early mixed dentition and may cause a variety of pathological disturbances to the developing permanent dentition. Early diagnosis and prompt treatment is necessary for prevention of deleterious effects on dentoalveolar structures. However, the time of intervention is the most crucial factor governing the outcome of surgical management of hyperdontia. The aim of this case report is to share knowledge about management of such cases, which might assist the clinician in decision-making in cases of impacted supernumerary teeth.

**How to cite this article:** Gupta S, Marwah N. Impacted Supernumerary Teeth–Early or Delayed Intervention: Decision Making Dilemma?. Int J Clin Pediatr Dent 2012;5(3):226-230.

## INTRODUCTION

A pedodontist is frequently confronted with dentoalveolar surgical problems in children, such as impacted teeth. Management of impacted teeth in children demands not only technical skill but also knowledge of eruption status, growth and development aspects of adjacent teeth and soft tissues. The following pathological sequelae have been noted to be associated with tooth impaction: Dentigerous cyst, odontogenic keratocyst, adenomatoid odontogenic tumor, calcifying epithelial odontogenic (Pindborg) tumor, odontogenic myxoma, ameloblastoma, ameloblastic fibroma, ameloblastic fibrous odontoma, ameloblastic fibrosarcoma, external/internal resorption of impacted tooth, external root resorption of adjacent teeth, transmigration, referred pain and periodontitis.^1-3^ A number of factors need to be evaluated before an impacted tooth is extracted, i.e. patient's age, dental status of adjacent teeth. (including periodontal, endodontic and operative status, shape, resorption), dental status of the impacted tooth, occlusal relationship and arch length.^1,4^ Complications that may be associated with surgical removal of impacted teeth include periodontally compromised adjacent teeth, damage to adjacent teeth, root fracture, neuropathy, sinus involvement and osseous defect.^5^ Thus, clinician should consider only those treatment goals for impacted teeth that minimize injury to the dentition and periodontium.^6^

Hyperdontia is a mammalian developmental anomaly characterized by presence of extra teeth in addition to the teeth of normal eruption series.^7^ Supernumerary teeth are considered to be one of the most significant dental anomalies affecting the primary and early mixed dentition and are considered a multifactorial inheritance disorder originating from hyperactivity of dental lamina.^8^ Based on location in the dental arch, a supernumerary tooth can be categorized into three types: Mesiodens, distomolar or paramolar and have a variety of forms, i.e. conical, tuberculate, supplemental or odontoma type.^9^

The incidence of supernumerary teeth is about 0.15 to 3.8% on a population basis^8,10^ with a predilection of 90 to 98% in maxilla, where the most favored site is premaxilla.^10,11^ Supernumerary teeth may erupt, stay impacted, appear inverted and assume an ectopic position or an abnormal path of eruption.^11^ They may remain in position for many years without clinical manifestations, either pathologic or orthodontic and at the same time can be associated with impaction, delayed eruption of adjacent teeth, crowding, midline diastema, cystic formation, root resorption of adjacent teeth, etc.^9^

Unless they are diagnosed early and managed properly, supernumerary teeth in maxillary anterior region may cause a variety of pathological disturbances to the developing permanent dentition.^12^ Thus, early diagnosis, evaluation and appropriate treatment is essential. Once the presence and position of supernumerary tooth is established, the desirability of surgical removal must be established. However, controversies exist regarding the optimum timing for surgical removal of supernumerary tooth. The aim of this case report is to share knowledge about management of such cases, which might assist the clinician in decision- making in cases of impacted supernumerary teeth.

## CASE REPORTS

### Case I

**Fig. 1:**
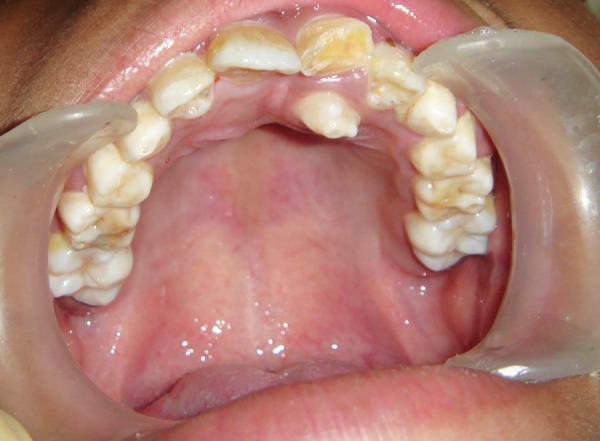
Intraoral photograph showing palatally erupted supernumerary tooth (case I)

**Fig. 2:**
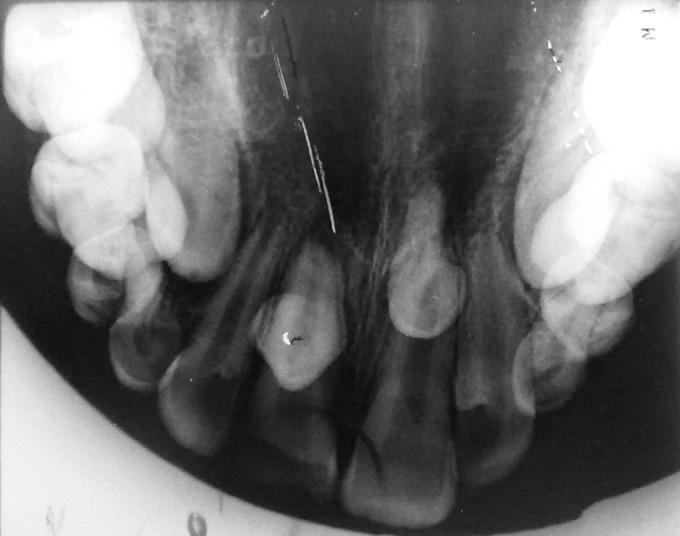
Maxillary occlusal radiograph revealing two supernumerary teeth (case I)

**Fig. 3:**
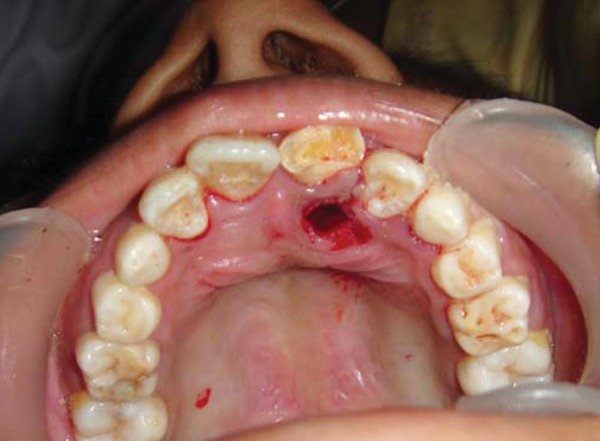
Intraoral view after removal of palatally erupted supernumerary tooth (case I)

**Fig. 4:**
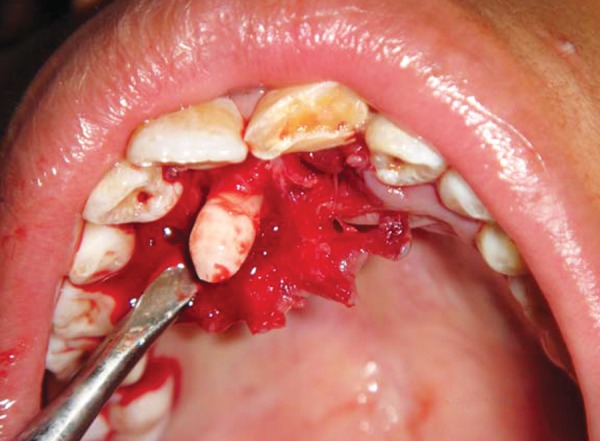
Intraoral photograph showing removal of impacted supernumerary tooth (case I)

An 11-year-old male child reported to the Department of Pedodontics and Preventive Dentistry, with a chief complaint of an extra tooth. The intraoral examination confirmed the patient's chief complaint and an erupted supernumerary tooth, located palatally, tuberculate in shape was located (Fig. 1). Radiographic examination revealed the presence of two supernumerary teeth, one erupted and one impacted, both in normal vertical alignment (Fig. 2). The medical history of the child was nonsignificant and he was born to non- consanguineous parents. The treatment plan involved surgical removal of both the mesiodentes after taking parent consent. Prior to surgical procedure, complete hematological investigations were performed to rule out any possible complications. The child was administered local anesthesia (greater palatine and nasopalatine nerve block). The palatally erupted mesiodens was extracted first (Fig. 3) and a full thickness palatal flap was raised using a mucoperiosteal elevator. The impacted tooth was exposed, luxated out of its socket and removed (Fig. 4). Hemostasis was achieved and the flap was replaced back and sutured with nonresorbable black silk suture. Postsurgical instructions were explained to the patient and he was kept on analgesic and antibiotic coverage. Patient was instructed to maintain a good oral hygiene using a soft bristle toothbrush and chlorhexidine mouthwash twice daily. The recall visits was scheduled for 1 week for suture removal and evaluation of healing followed by a 6 monthly recall pattern for continued observation. The patient is on follow-up for the last 1 year.

### Case II

**Fig. 5:**
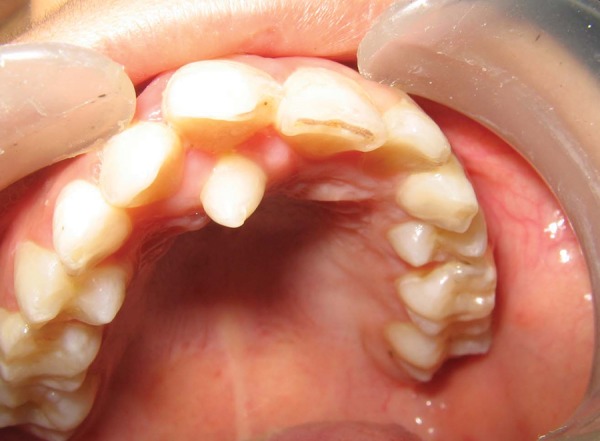
Intraoral photograph showing palatally erupted supernumerary tooth (case II)

**Fig. 6:**
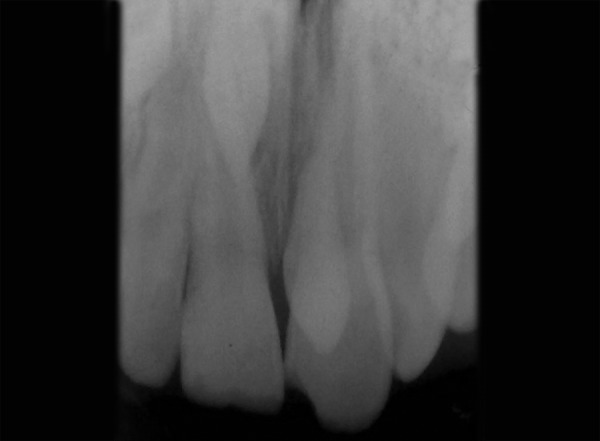
Intraoral periapical radiograph revealing two supernumerary teeth (case II)

**Fig. 7:**
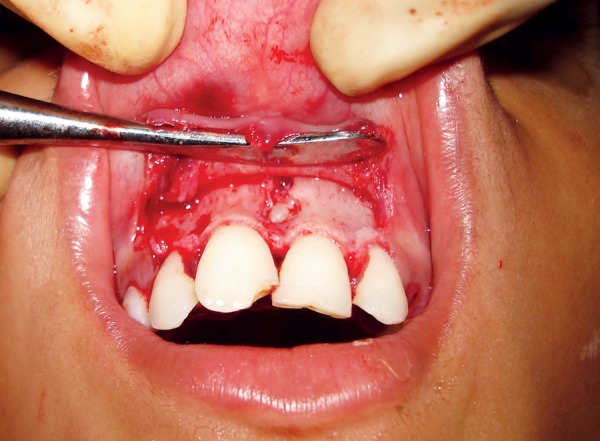
Incisal tip of impacted supernumerary tooth after raising labial mucoperiosteal flap (case II)

**Fig. 8:**
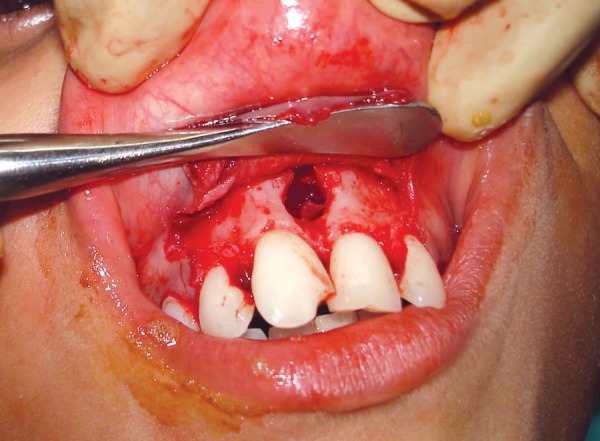
Empty socket after removal of impacted supernumerary tooth (case II)

**Fig. 9:**
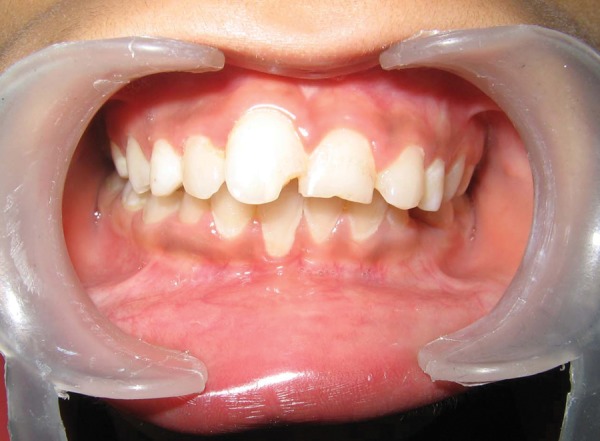
Postoperative photograph showing uneventful healing (case II)

A 9-year-old female patient presented at the Department of Pedodontics and Preventive Dentistry, with a chief complaint of a sharp pointed tooth inside oral cavity which irritated her tongue. Intraoral examination revealed an erupted conical mesiodens in the anterior palate (Fig. 5). Radiographic examination revealed two mesiodentes, one erupted and one impacted, both conical in shape (Fig. 6). Treatment plan involving surgical removal of both mesiodentes was explained to the parents. Complete hemogram and parental consent was obtained prior to initiation of the surgical procedure. The case demonstrated the possibility of occurrence of the impacted mesiodens in a more vestibular location, as detected by SLOB technique. The palatally erupted mesiodens was first removed and labial approach for surgical removal of impacted mesiodens was planned. For this, a labial apically repositioned flap was raised using a mucoperiosteal elevator (Fig. 7). The bone overlying the crown of the tooth was removed using carbide bur and saline spray. The tooth was exposed and removed from its socket (Fig. 8). The flap was replaced and sutured with black silk suture and postoperative instructions were given to the patient. The patient is on follow-up and healing is uneventful (Fig. 9).

### Case III

**Fig. 10:**
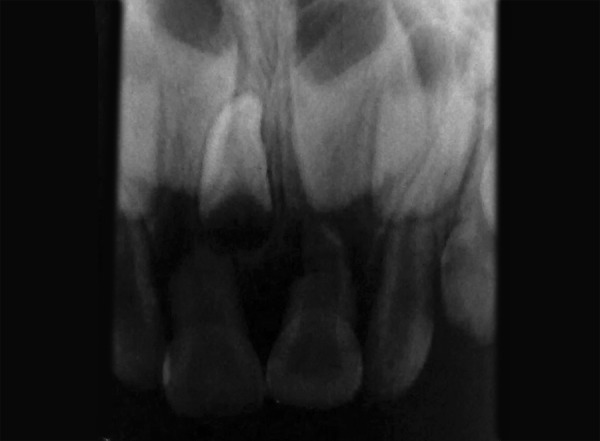
Intraoral periapical radiograph revealing inverted impacted supernumerary tooth (case III)

A 5-year-old child came to the Department of Pedodontics and Preventive Dentistry, accompanied by her mother. The mother informed that her elder child had an extratooth in her mouth which had to be extracted so she brought this child for check up to rule out any such problem in her also. Intraoral examination revealed primary dentition with end on molar relationship. Intraoral periapical radiograph of premaxillary region revealed an inverted mesiodens with a conical appearing crown and incomplete root formation (Fig. 10). The extraction of supernumerary teeth was delayed due to incomplete root formation and owing to the position of the teeth as surgical retrieval of supernumerary tooth would have damaged the erupting tooth buds. The patient was kept on follow-up and evaluated every 3 months to check any dental malformations. The extraction was then carried out after 2 years when the central incisors erupted and there was no possibility of damaging the surrounding tooth structures during surgical removal of supernumerary tooth. The patient is normal and all the wounds have healed uneventfully justifying the delayed intervention plan.

### Case IV

**Fig. 11:**
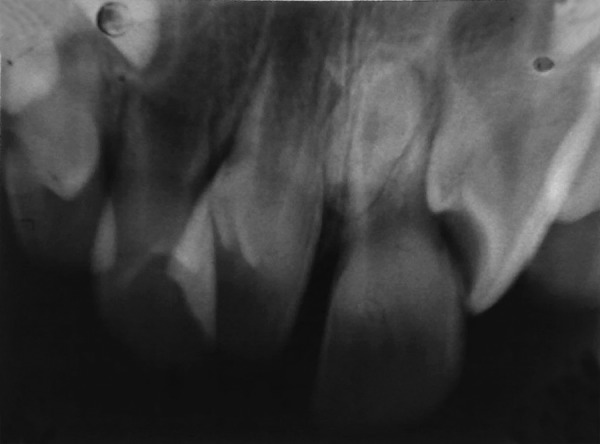
Intraoral periapical radiograph revealing impacted supernumerary tooth (case IV)

A 7-year-old female child reported to the Department of Pedodontics and Preventive Dentistry, accompanied with her parents, who were anxious about delay in eruption of an upper front tooth. Intraoral examination revealed missing permanent left maxillary central incisor. Intraoral periapical radiograph revealed an impacted conical mesiodens with inverted alignment (Fig. 11). Root apices of the permanent central incisors were not closed. The surgical intervention was delayed till the completion of root closure of central incisors. The patient was on follow-up for 1 year following which the supernumerary was extracted surgically after central incisor had obtained root closure. The healing is uneventful and the central incisors have now erupted in the oral cavity.

## DISCUSSION

Mesiodens is the most commonly impacted tooth in pediatric patients.^13^ Clinically, the presence of supernumerary tooth should be suspected if there is a delay in eruption of maxillary central incisors. Discrete appearance of any eruption or occlusal problem is worth investigating, to diagnose the occurrence of unerupted supernumerary tooth as etiologic factor.^9^ The first phase of management of impacted supernumerary teeth is localization and identification of complications associated with them.^14^ The recognition of supernumerary teeth on radiographs does not present problem, but in early stages prior to calcification, they may have cyst like appearance. Care must be taken to avoid misinterpretation of overlapping cusps and other anatomical landmarks which may simulate supernumerary teeth.^15^ Localization of the impacted tooth is one of the more challenging and unpredictable aspects of surgical exposure of the impacted tooth.^16^ A series of periapical radiograph taken using paralleling techniques gives the most detailed assessment. Clark pioneered this technique in 1910 using the cone head shift in horizontal plane. By 1952, Richards added a vertical shift to this process and supplemented the ability to locate the tooth.^17^

The inverted conical from of supernumerary tooth is frequently associated with cystic lesions and can erupt into the nasal floor, becoming more difficult to remove with time.^18^ The tuberculate form rarely erupts, is frequently bilateral and interferes with permanent tooth eruption^3^ and thus removal of these teeth is recommended.^4^ Surgical removal is the treatment of choice to prevent clinical complications and also to treat an established complication. It is in the patient's best interest to have the tooth removed immediately on discovery unless a good reason for their retention can be found.^19^ However, parents are not always willing to go for a surgical procedure for a condition they are unaware of and which is not causing any immediate problem. The practitioner should emphasize that the untreated tooth may become cystic and expand considerably and undergo morphologic changes in a short period.^20^ Additionally, there is a possibility of ameloblastoma formation in the walls of the follicle.^21^ Thus, surgical removal of the impacted supernumerary teeth was planned in case I and II to prevent any such complications in the future and also because the patient's age was favorable in both the cases.

### Early Intervention

It is indicated as an approach which may result in spontaneous correction of an existing malalignment. If the mesiodens is detected and removed early, the impacted incisors may erupt into position without orthodontic treatment.^22^ Some authors feel that unless the supernumerary tooth is diagnosed as not causing any abnormality to the permanent dentition, early surgical intervention is preferred to take advantage of the spontaneous eruptive potential of permanent incisors and to prevent anterior space closure and midline deviation.^23^ Furthermore, with subsequent growth, the increase in vertical height of premaxilla will accentuate the difficulty of surgical access to the unerupted supernumerary, particularly of the inverted type.^11^ However, some of the possible disadvantages of early intervention^9^ may include damage to adjacent teeth resulting in loss of vitality and root malformation and inability of a young child to psychologically tolerate the surgical procedure.

### Delayed Intervention

The optimum time for surgical removal of an erupted maxillary anterior tooth is controversial. Some authors advocate immediate removal of supernumerary tooth following diagnosis of their presence, while others favor postponement of surgical intervention until the age of 8 to 10 years, when the root development of central and lateral incisors is complete.^10,11^ Thoma^25^ believed that if mesiodens is not interfering with eruption, its extraction should be delayed until roots of permanent central incisors have formed completely to avoid injury to developing root structure. The best time for removal is at nearly 8 to 9 years of age, when the behavior of child is much easier to manage and the type of anesthesia can be less invasive, thereby reducing the trauma and anxiety associated with the surgery.^26^ Also, the clinician should ensure that the adjacent permanent central incisors have completed one-half to two-third of their root development, which decreases the likelihood of injury to these teeth.^13^ Surgical removal of supernumerary tooth in primary dentition is usually not recommended, because of the risk of displacing permanent tooth during operation.^27^ Keeping the above factors in mind the delayed intervention was planned in case III and IV. But, some of the possible disadvantages of delayed intervention include^9,24^ diminished eruptive forces of adjacent teeth, loss of anterior arch space and midline shift.

Early diagnosis, establishment of position of the supernumerary tooth, dental status of surrounding tooth structure along with the knowledge of developing dentofacial complex is mandatory for the fabrication of treatment plan using early or delayed intervention. Thus, all these factors must be taken into consideration before surgical intervention in cases of hyperdontia.
